# Comments on “Efficacy and safety of adjunctive corticosteroids in the treatment of severe community-acquired pneumonia: a systematic review and meta-analysis of randomized controlled trials”

**DOI:** 10.1186/s13054-023-04619-y

**Published:** 2023-09-06

**Authors:** Ming-hao Luo, Zengyi Wan, Guo-wei Tu, Zhe Luo

**Affiliations:** 1 Cardiac Intensive Care Center, Zhongshan Hospital, Fudan University, Shanghai, 200032 China; 2 Shanghai Medical College, Fudan University, Shanghai, China; 3 Weill Cornell Medicine, New York, USA

Dear editor,

We read with great interest the article by Wu et al., in which they studied the efficacy and safety of adjunctive corticosteroids in severe community-acquired pneumonia [1]. The authors ought to be congratulated for such an updated review. However, we have concerns regarding the conclusion of analysis.

## Heterogeneity among different studies

The authors pooled results from seven studies from 1993 to 2023 and used the *I*^2^ statistic to assess the heterogeneity. The authors reached the conclusion that “low heterogeneity in most outcomes” was observed based on low *I*^2^ estimates. However, tests for heterogeneity using the *I*^2^ statistic is often underpowered, especially with a small number of included study; it is thus insufficient to conclude that the studies have low heterogeneity based on the *I*^2^ statistic alone.

In addition, a close examination of the seven included studies would reveal several potentially important sources of heterogeneity. First, the definition of comparison for each randomized controlled trial (RCT) is different as the standard of care has changed significantly over the past 30 years. The increasing prevalence of antimicrobial resistance leading to varying choices of antibiotics, adoption of high flow nasal cannulation, and changing ventilation strategies are some examples [2–4]. Second, different regimens of corticosteroids were administered in each RCT, which should result in significant variability among the studies included. Third, different patient populations were included in each RCT. For example, Torres et al.’s study focused only on patients with C-reactive protein (CRP) > 150 mg/L at admission while other studies did not exclude patients based on their CRP levels [5]. The pooled estimates should thus be interpreted with caution and consideration for the qualitative differences among the studies. To account for the low power of the *I*^2^ statistic, it may be of interest to still conduct an exploratory analysis of the potential sources of heterogeneity using meta-regression.

## Data inputs

The authors performed a number of interesting subgroup analyses. With the importance of Dequin et al.’s study on the pooled estimates, we noticed an error in data entry. In Dequin et al.’s study, intravenous hydrocortisone was administered “200 mg daily for either 4 or 7 days as determined by clinical improvement, followed by tapering for a total of 8 or 14 days,” not “200 mg daily for either 4 or 8 days” [6]. It should thus be included in the tapering subgroup and the > 8 days of treatment subgroup in the subgroup analysis. Further, the mortality outcomes from the included studies were not consistent and did not always match the primary outcome of this meta-analysis (i.e., 30-day all-cause mortality) (Table 1).

**Table 1:** Mortality type and CRP reported in the included study

References	Year	Reported mortality	How CRP was integrated
Dequin et al. [6]	2023	28-day and 90-day mortality	Reported in baseline, in subgroup analysis (threshold: 15 mg/dL)
Meduri et al. [7]	2022	All-cause mortality at days 60, 180 and 365	Not indicated
Torres et al. [5]	2015	Death within 72 and 120 h of treatment, in-hospital mortality	Reported in baseline; clinical outcomes were compared between patients with a still high CRP (> 130 mg/L) at day 7 and those who were not
Sabry et al. [8]	2011	Mortality by day 8	Reported in baseline and was compared on day 2, 4, 6 and 8
El-Ghamrawy et al. [9]	2006	Reported mortality rate without specifying the type	Not indicated
Confalonier et al. [10]	2005	Mortality by day 8, ICU mortality, hospital mortality and 60-day mortality	Reported in baseline and was compared on day 1, 2, 3, 6 and 8
Marik et al. [11]	1993	Reported mortality rate without specifying the type	Not indicated

*CRP* C-reactive protein, *ICU* intensive care unit

## Subgroup analysis

The authors conducted subgroup analysis based on pre-defined criteria and found that mortality benefits were consistently observed in most of the subgroup analyses, particularly for patients aged 60 years or older, without initial septic shock, with ICU admission, use of hydrocortisone and receiving corticosteroid for a duration of ≤ 8 days and not undergoing corticosteroid tapering. However, it should be noted that some subgroups were derived from only one study, in addition to the point mentioned above that a few studies were misclassified. More importantly, the level of CRP was not considered in the subgroup analysis even if previous trials revealed its clinical significance. In a study conducted by Dequin et al., although overall mortality benefits were observed, subgroup analysis revealed no significant difference in the number of deaths among patients with a CRP of under 15 mg/dL (− 2.4 percentage points; 95% CI − 10.7 to 6.0) [6]. The use of CRP, whether as an inclusion criterion in future clinical trials or as part of subgroup analysis, needs to be emphasized (Table 1).

Overall, the published meta-analysis has offered important information regarding the rationale of corticosteroids in patients with severe community-acquired pneumonia. But the improvement in mortality, and the population in which corticosteroids would reveal the most benefits should be interpreted critically and reviewed with caution. Further studies are urged to offer more definitive answers.

## Data Availability

None.
